# Visual analytics tool for the interpretation of hidden states in recurrent neural networks

**DOI:** 10.1186/s42492-021-00090-0

**Published:** 2021-09-29

**Authors:** Rafael Garcia, Tanja Munz, Daniel Weiskopf

**Affiliations:** VISUS, University of Stuttgart, 70569 Stuttgart, Germany

**Keywords:** Visual analytics, Visualization, Machine learning, Classification, Recurrent neural networks, Long short-term memory, Hidden states, Interpretability, Natural language processing, Nonlinear projection

## Abstract

In this paper, we introduce a visual analytics approach aimed at helping machine learning experts analyze the hidden states of layers in recurrent neural networks. Our technique allows the user to interactively inspect how hidden states store and process information throughout the feeding of an input sequence into the network. The technique can help answer questions, such as which parts of the input data have a higher impact on the prediction and how the model correlates each hidden state configuration with a certain output. Our visual analytics approach comprises several components: First, our input visualization shows the input sequence and how it relates to the output (using color coding). In addition, hidden states are visualized through a nonlinear projection into a 2-D visualization space using t-distributed stochastic neighbor embedding to understand the shape of the space of the hidden states. Trajectories are also employed to show the details of the evolution of the hidden state configurations. Finally, a time-multi-class heatmap matrix visualizes the evolution of the expected predictions for multi-class classifiers, and a histogram indicates the distances between the hidden states within the original space. The different visualizations are shown simultaneously in multiple views and support brushing-and-linking to facilitate the analysis of the classifications and debugging for misclassified input sequences. To demonstrate the capability of our approach, we discuss two typical use cases for long short-term memory models applied to two widely used natural language processing datasets.

Video abstract.

**Supplementary information:**

**Supplementary information** accompanies this paper at 10.1186/s42492-021-00090-0.

## Introduction

Interpretability is a major issue faced by modern machine learning (ML). Although some ML techniques, such as decision trees or logistic regression, tend to be easy to interpret, they often do not handle complex prediction problems well. Others, such as neural networks, can address much more difficult problems but usually fail to deliver an easily interpretable solution.

In particular, interpretability is a major issue in deep neural networks (DNNs) [1]. These models often have up to millions of learnable parameters, which make it difficult for humans to interpret the calculations. The black-box behavior has halted the use of deep learning in applications such as medicine, robotics, and finance. Overcoming this problem has become a major goal in ML research during the past few years [2].

A popular approach to achieving interpretability is visualization [2, 3] because it allows users to make sense of large, high-dimensional, and temporal data from ML. In particular, visual analytics [4] is useful for interactively exploring data in multiple coordinated views and providing important insight. Some of the challenges that visualization can address include explaining which input features the model has learned to recognize [5–7] and how the activation vectors produced by hidden layers transform the input data into a more abstract representation, making predictions easier [8–10].

However, few techniques tackle the interpretability of hidden states in recurrent neural networks (RNNs) [11, 12]. These models are designed to address ML problems with temporal input data, such as financial data or text-based datasets. They do so by employing *recurrent layers*. These layers maintain an internal vector, i.e., a *hidden state*, whose values are updated every time a new data element is fed into the network, working as a memory that keeps information from previous time steps to build the final activation vector once the entire sequence has been processed. Recurrent layers introduce multiple interpretability problems that are absent in other types of layers, such as convolutional or fully connected layers.

**Fig. 1:**
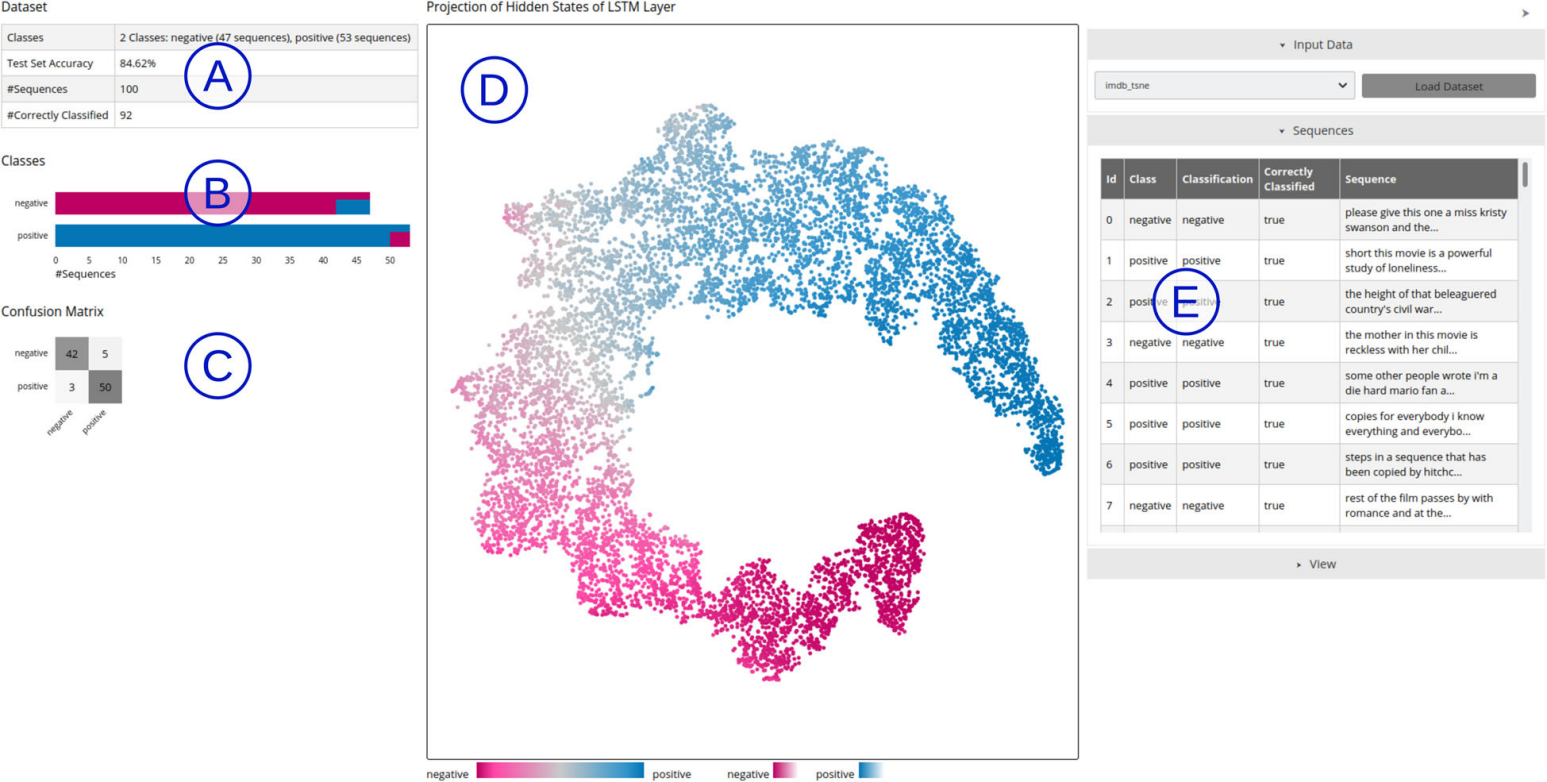
The main view of our visual analytics approach applied to a model trained on the IMDB dataset. Some general information about the dataset is shown on the panels (A) to (E). (**A**): The number of input sequences, the classes, and how well the sequences were classified; (**B**): Visualizations of the different classes to compare the number of input sequences that were correctly classified; (**C**): This information is also visible in the form of a confusion matrix; (**D**): At the center, the projection of all hidden states produced by the network for all input sequences is visible; (**E**): On the right side, a list of all sequences allows the selection of a sequence for further exploration. Underlying data source: IMDB as available in Keras [13]

**Fig. 2:**
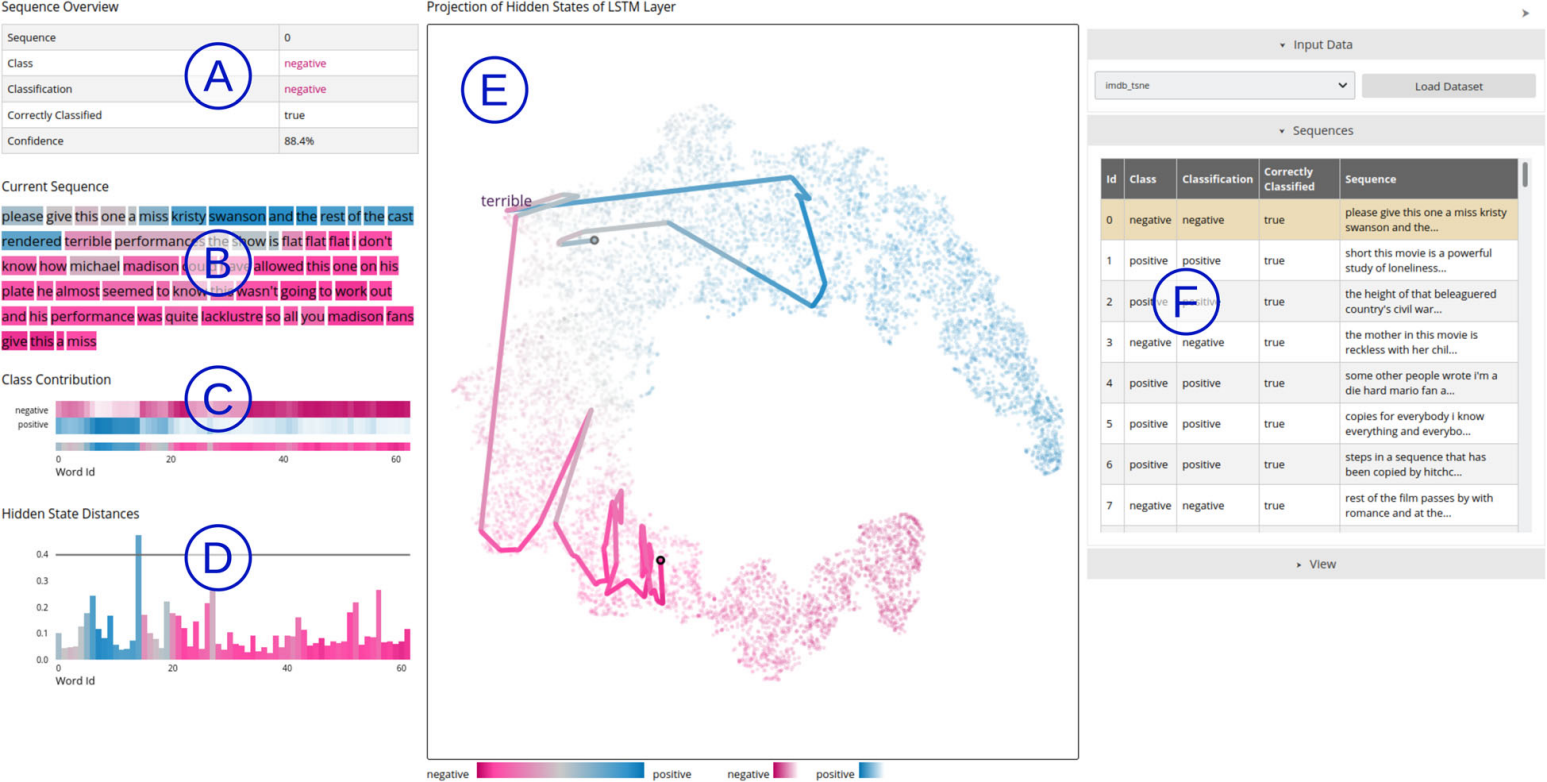
Detailed view for one selected sequence from the IMDB dataset. Different information and interactive visualizations are shown on panels (A) to (D). (A): The sequence ID and information about the classification; (B): The input sequence with each word colored according to its expected prediction (EP), i.e., what output the model would produce if that word was the last in the sequence; (C): A heatmap matrix displaying how the model’s prediction evolves during the sequence processing for each class (top) and overall (bottom); (D): A histogram of the Euclidean distances between the hidden states of the input sequence; (E): The center highlights in the projection the hidden states produced by the sequence, giving insight into how the hidden state evolves over the sequence processing. It is possible to define a threshold for distances to show words in the projection resulting from a larger change in the hidden state; this threshold is also visible as a horizontal line in the histogram; (F): On the right side, the currently selected sequence is highlighted in a list. In the example used for these visualizations, we note how the model first believed the sequence to be classified as positive during the first few time steps of the input sequence. However, as it obtained more information about the input, it changed its output to a negative value. Underlying data source: IMDB as available in Keras [13]

In this paper, we discuss these challenges and why existing techniques do not address them entirely. We also introduce a visual analytics system to improve the interpretability of RNNs, uncovering insight into how the hidden states encode information and how these values evolve throughout the input process. In particular, we focus on natural language processing (NLP) tasks, one of the main applications of an RNN. As an example, we apply our tool to long short-term memory (LSTM) models. Our visual analytics system for the binary classification of input sequences is shown in Figs. 1 and 2. We also discuss some use cases of how visual analytics can better explain the decision process of the model and thus answer a number of interpretability questions.

This paper is an extension of our previous work [14]. We extended our approach by implementing an interactive visual analytics system that facilitates the exploration of the classification process through brushing-and-linking [15] in multiple coordinated views [16]. The system supports the detection of correctly or incorrectly classified sequences and the process of debugging misclassified sequences to identify why an incorrect prediction was performed and which words contributed to such a prediction. We provide additional information about the Euclidean distances between the hidden states and the words that trigger larger changes. Further, we added visualizations that provide general information about the classification results of the underlying model to provide an initial quality assessment of the classification of all input sequences.

In addition, a supplemental video demonstrates how to use our visual analytics tool. In this video, we show how our tool can answer interpretability questions on models trained for sequence classification. We also provide the source code for our system [17, 18].

## Related work

Visualization and visual analytics provide interpretability to DNNs at several levels [2]. For instance, some techniques have been specifically designed for an analysis of classification models. For example, ClaVis [19] is a visual analytics system used to compare multiple classifiers. Other approaches can be used to explain which input features were taken into account by the model to formulate a prediction [5, 20] and how the decision process of the model transforms the input data into a more abstract representation that facilitates the prediction [2, 3]. In particular, to highlight the differences between the activations produced by elements of different classes, activation vectors of the models can be visualized, as an example, by employing dimensionality reduction [8] and heatmap matrices [9].

Although these methods manage to explain which input features impact the classification, they offer little insight into how the model uses the input features to build predictions. To address this problem, more recent techniques have focused on an analysis of the internal activation vectors produced by the hidden layers of the network [2, 3]. Neural networks process inputs by sequentially applying the operation defined by each layer on the input data, generating an activation vector that serves as the input for the next layer. Activation vectors can be seen as a more abstract representation of the input data. They iteratively transform the input data in a way that makes it easier to conduct the classification.

For a deep model to perform well, its hidden layers should produce data representations whose classes are more distinguishable. Ideally, elements from the same class should generate closely related activation vectors in the final layers, whereas elements from different classes should generate different activations. When this is not the case, it is an indication that the model is underperforming and may not be suitable for that application.

Multiple studies have used visualization techniques to analyze activation vectors [8, 9]. They employ techniques such as dimensionality reduction [8] and heatmap matrices [9] to highlight the differences between the activation vectors produced by elements of different classes. By doing so on multiple layers, they can show the decision process of the model and how the input representation evolves layer-by-layer. It also allows the user to identify classes that may be harder for the model to distinguish.

An activation vector analysis is as important for an RNN as it is for other architectures, and the techniques above can also improve the interpretability of these models. However, RNNs have extra complexity owing to the presence of hidden states, i.e., internal memories that store information from previous time steps in the input sequence. When a recurrent model reads an input sequence, its internal hidden states are updated at each time step, generating an activation vector after the entire sequence is processed. Such an analysis is even more challenging when working with models trained for NLP tasks [21]. In a text classification task, some of the input words may have a much larger impact on the hidden states and the final prediction than other words. In addition, some words may produce information that must be stored in the hidden states for a longer period of time than other words. The natures of RNNs and NLP provide new challenges that are not addressed through techniques developed only with an activation vector analysis in mind.

Some techniques have previously focused on improving the interpretability of RNNs trained for NLP tasks. For instance, LSTMVis [22] allows the user to identify patterns in the hidden states when processing a sequence, such as multiple-input words that produce a similar hidden state. Likewise, RNNVis [23] employs a hidden state cluster visualization to correlate groups of similar input words with hidden state configurations.

Although these tools can display patterns in the hidden state and relate them to particular inputs, they do not address open interpretability issues. In particular, to the best of our knowledge, there are no techniques that have addressed the problem of visualizing how hidden state configurations are distributed in a high-dimensional space and how different regions of this space correlate to different prediction values. In addition, they did not evaluate the impact of each input step in the hidden state configuration or consequently in the final prediction. In this paper, we aim to discuss these challenges and introduce a set of techniques that can resolve them.

## RNN interpretability challenges

The temporal nature of an RNN introduces new interpretability challenges when compared to other DNNs. Below, we list three interpretability challenges that, according to our research, must be addressed to achieve better interpretability with an RNN. This is not meant to be exhaustive. Recurrent networks share several characteristics with other architectures, which mean that there is a strong intersection between the interpretability issues present in RNNs and those in other models. Herein, we focus on issues specific to RNNs.

Input-to-hidden-state correlation: When an RNN processes a sequence, it has an initial hidden state vector in each recurrent layer. When the layer receives the next element from the input sequence, this vector is updated according to the information extracted from that piece of information. This behavior allows the model to combine information from previous and future time steps to build a final activation vector that, ideally, should contain the features needed for the prediction task. Hence, the same input step (e.g., the same word in an NLP model) can generate a vastly different hidden state depending on the words that came before it. In addition, the impact of such an input on the hidden state may differ, since the initial time steps usually have a higher impact owing to the lack of information of the model at this point. The analysis of the correlation between the input values and their impact on the hidden state is a key element in improving the interpretability.

Hidden state space analysis: Hidden states are an abstract, high-dimensional representation of the input sequence. However, unlike activations, they are built iteratively during the processing of the input. The layer updates its hidden state at every time step. We can interpret the space of all possible hidden state configurations as a high-dimensional space within which the subsequent layers apply a classification. Analyzing this space and understanding how different configurations are spread over this space are important for increasing the interpretability of the RNNs and answering questions such as whether there is strong class confusion in the configurations and how they evolve during the sequence processing.

Hidden-state-to-output correlation: A model with a good performance should produce hidden states that are easily distinguishable from the hidden states of the opposing classes, particularly in deeper layers. Because the initial time steps may not hold sufficient information to distinguish between classes, this separability must be built through the input processing. An important interpretability challenge of an RNN is to identify how intermediate hidden states, created during sequence processing, correlate to the prediction, and how this prediction changes when the RNN receives new time steps.

## Methods

Our visual analytics technique addresses the three challenges discussed in the previous section . Our interactive approach consists of multiple views [16], allows brushing-and-linking [14], and uses a visual analytics approach [4]. We show different visualizations for different aspects of the classification, where users can interact with the data provided for analysis. Our system consists of an overview of all classification data (Fig. 1) and a more detailed presentation of individual sequences (Fig. 2).

**Fig. 3:**
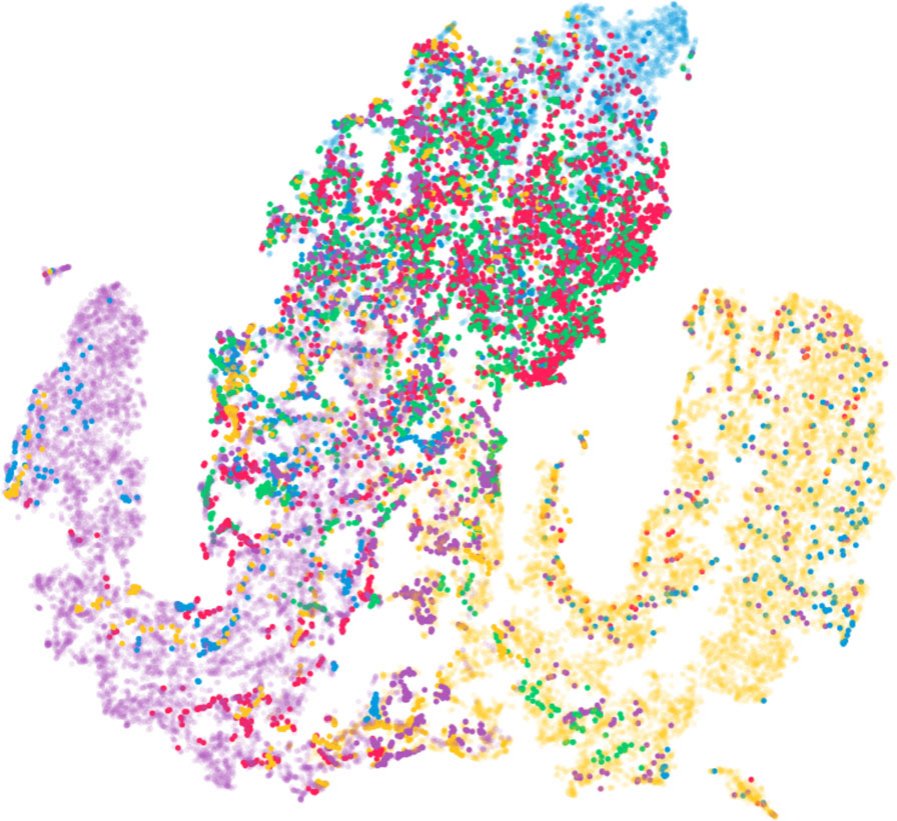
Projection for the Reuters dataset. The data points of the sequences are colored according to their actual class instead of the classification result. In addition, points that belong to correctly classified sequences are more transparent. This visualization helps identify areas where hidden states generate possibly incorrect model outputs. At the top, most sequences were classified as *money-fx* (blue) instead of *crude-oil* (green) or *grain* (red). Underlying data source: Reuters [25]

The overview provides (1) some general information regarding the dataset and its classification results [Fig. 1(A-C)], (2) the projection of the hidden states produced for different time steps and input sequences for the entire dataset [Fig. 1(D)], and (3) a table with the properties of all sequences, allowing the user to select one sequence for further analysis [Fig. 1(E)].

**Fig. 4:**
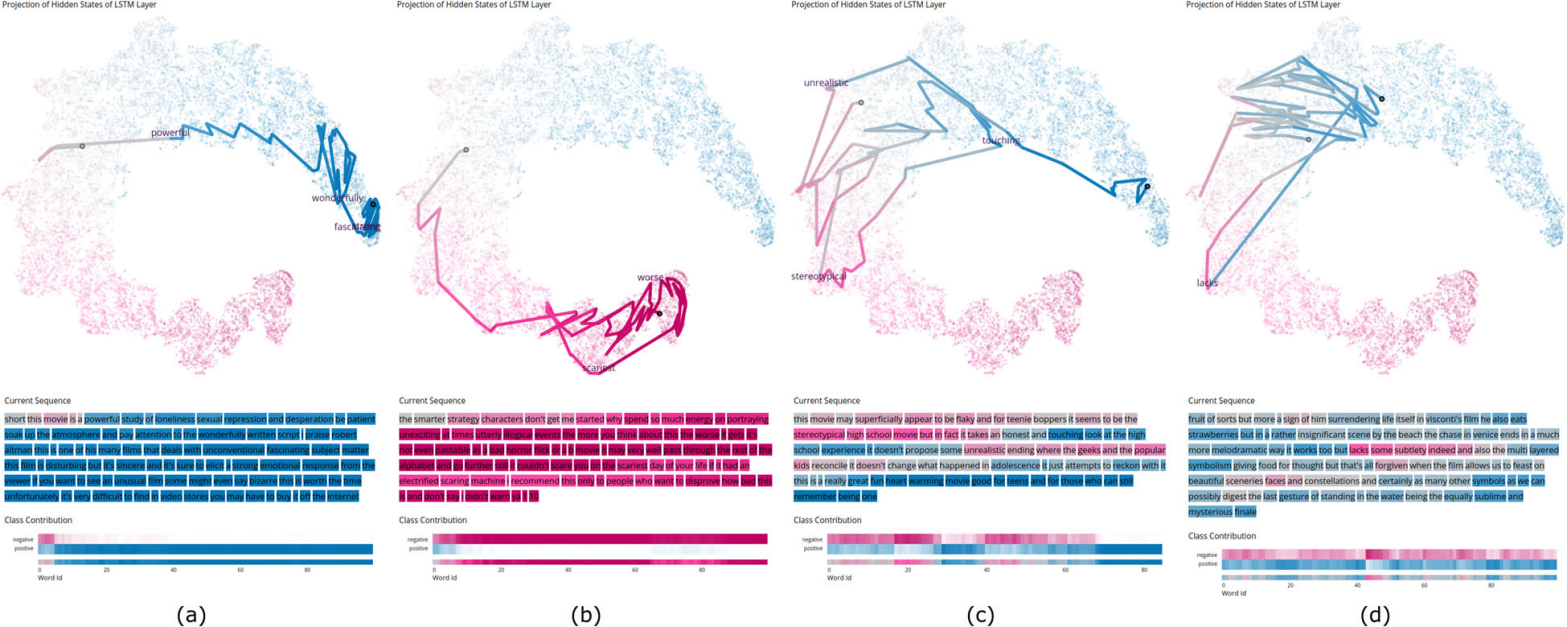
Different examples of correctly classified sequences from the IMDB dataset. From left to right: (**a**): Typical example of a positively classified movie (blue). The first word that clearly classifies the sequence as positive is ‘powerful’. Afterward, no word suggests that the movie might have been negatively rated. The words ‘wonderfully’, ‘strong’, and ‘fascinating’ result in particularly larger jumps in the hidden state space; (**b**): Typical example of a negatively classified sequence (pink). Right from the beginning, this review was classified negatively; (**c**): The input starts with several negative words (pink), making the model alternate between hidden states with high and low EPs. Toward the end of the review, the intent of the writer becomes clear and the model settles for a positive output (blue); (**d**): A positively classified movie with uncertainties. At the beginning and end, there are more indications of a positive rating (blue). However, most vocabulary feels more neutral than highly positive. In the middle, there is also a negative statement (pink). Underlying data source: IMDB as available in Keras [13]

**Fig. 5:**
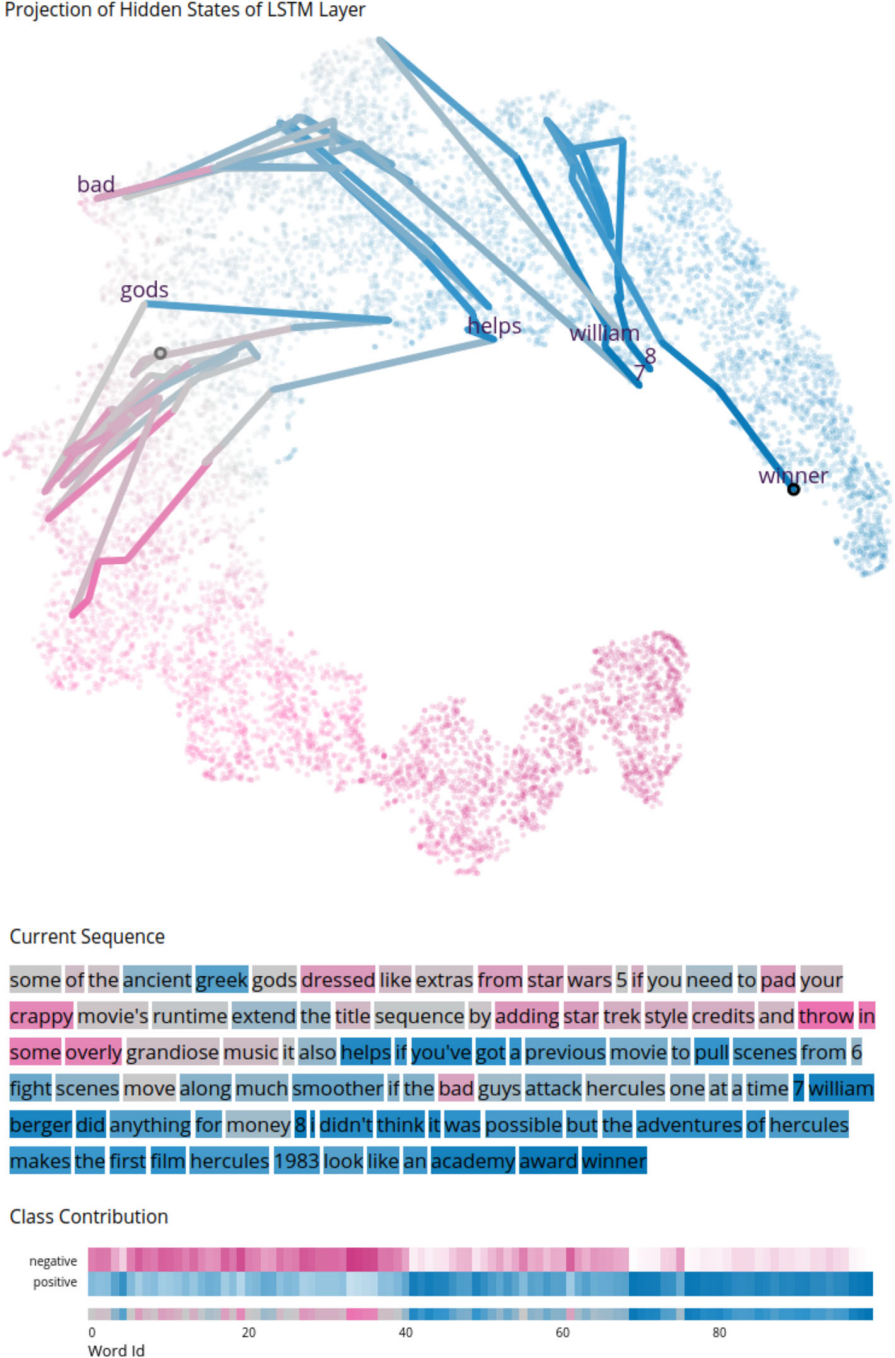
Example of a wrongly classified sequence from the IMDB dataset. For the first part of the sequence, the classification is rather negative (pink). Only in the second half of the sequence does the classification become positive (blue). The last words in particular contribute to the incorrect classification, where the name of an actor appears, and afterward, the words refer to another film that was rated better compared to the current one. Underlying data source: IMDB as available in Keras [13]

**Fig. 6:**
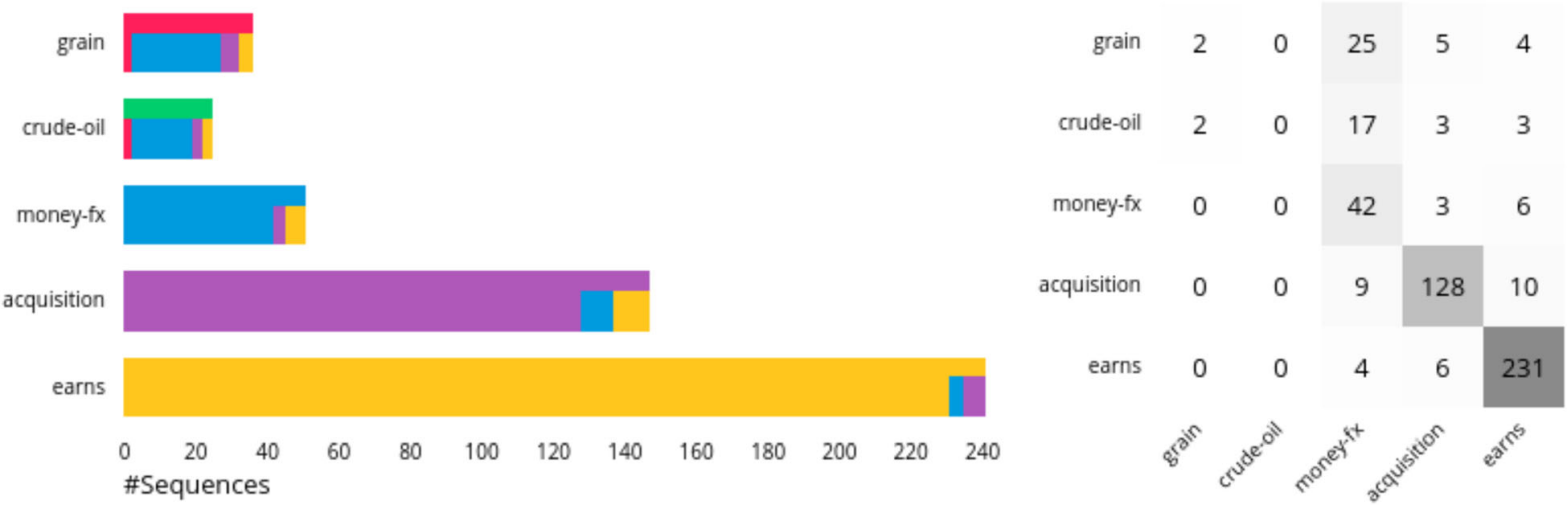
Classification result in the form of stacked bar charts (left) and a confusion matrix (right). The visualizations show that there are many more sequences available for the classes *earns* and *acquisition* compared to the others. In addition, it is clear that sequences of the classes *earns*, *acquisition*, and *money-fx* were well classified, whereas the model had problems with *grain* and *crude-oil*. Underlying data source: Reuters [25]

Sequence-based visualizations are composed of multiple coordinated views, i.e., (1) some general information about the classification of the current sequence [Fig. 2(A)], (2) a visualization of a user-specified input sequence colored by the expected output for every partial sequence [Fig. 2(B)], (3) highlighting of the trajectory followed by the hidden states produced through the selected input [Fig. 2(E)], (4) a heatmap matrix displaying how the expected output evolves through the processing of the input sequence selected by the user [Fig. 2(C)], (5) a histogram showing the Euclidean distances between the hidden states of the input sequence [Fig. 2(D)], and (6) a table where the current sequence is highlighted [Fig. 2(F)].

To build these visualizations, we first trained the model (next section) and extracted the hidden states produced in each time step for all test data. Thus, we are left with a dataset of $H=T \times N$ hidden states, where $T$ is the number of time steps and $N$ is the number of test sequences. Each hidden state ${h}_{i}\in H$ is a vector. For each of these configurations ${h}_{i}$, we calculated the EP ${p}_{i}$. An EP  is the output produced by the model if the hidden state ${h}_{i}$ were the last time step of the sequence fed into the model. The EP tells us how the model classifies the input until that moment, and consequently how later time steps modify the model decision. We describe our visualization technique in more detail below.

Classification overview: When loading a new dataset, an overview of the classification quality of the available sequences is presented [Fig. 1(A-C)]. Herein, we used two different visualization approaches to show the effectiveness of the classification for different classes. (1) A stacked bar chart shows the number of sequences for each class in the dataset and their classification results. Each class has a unique color. The lengths of the bars indicate the number of sequences. On the top, the color of the actual class is visible, and at the bottom, a stacked bar shows the classification of the sequences. (2) A confusion matrix quantitatively reports the classification results. Both visualizations show the same information in different ways. We decided to include both of them because the confusion matrix represents the data in a conventional manner, and the stacked bar chart provides a quick overview of the classification results and links to the other views by using the same color encoding.

Hidden state projection: By projecting the entire collection of hidden states $H$ into a 2-D space using t-distributed stochastic neighbor embedding (t-SNE) [24] (or another projection method), we can analyze how the space of hidden state configurations is shaped (second challenge from the previous section). Herein, we have a set of hidden states ${h}_{i}\in H$ that consist of the hidden states of each sequence for each time step, where each ${h}_{i}$ is represented as a vector. We then use dimensionality reduction on all hidden states ${h}_{i}$ to project them into 2-D for visualization. The dimensionality of each hidden state depends on the number of hidden units that can be individually defined for LSTMs. In our experiments, we used t-SNE; however, other projection techniques can also be applied. A discussion on which projection technique or parameter configuration leads to the best visualization for a particular model is beyond the scope of our paper. To ensure the reproducibility, we made our code publicly available [17]. By coloring the data points according to their corresponding EP, we can analyze how different regions in the hidden state space correlate to the level of confidence that the model has with a given prediction. Figure 1(D) shows an example of this visualization for a binary classification. Because the network has a single softmax output, our color encoding comprises a range from zero to 1. For a binary classification, a prediction of zero (a completely negative review) is denoted by pink, and an output of 1 (a completely positive review) is denoted by blue. A linear color gradient is used from the first to the second color over gray (where the prediction is uncertain).

Although the data points are colored according to the EP by default, it is optionally possible to differentiate between correctly and incorrectly classified sequences using transparency. In addition, all data points belonging to a sequence can be instead colored by the actual class of a sequence (Fig. 3). This helps identify regions of hidden states in which the classification is rather certain or may have difficulties.

Input visualization: We visualize each time step of the input sequence, with each word colored according to the EP produced by the model after reading that word [Fig. 2(B)]. Hence, the user obtains insight into which time steps are more important for classification and on how the EP evolves over the processing of the sequence, addressing the first challenge from the previous section.

Visualizing the expected evolution of the prediction: The EP of multi-class classification models is multidimensional, and thus cannot be encoded by a single color. A usual option is to color the data points according to the label with the highest confidence; however, doing so implies that we will lose valuable information about the strength of such confidence. To avoid this problem, we add a supportive heatmap matrix visualization [Fig. 2(C)], which displays the evolution of the EP vector over the sequence processing (the first challenge from the previous section). Each row displays one possible class, and each column denotes a single time step within the sequence. The color of each square represents the EP of the model for that class at that time step. With our tool, opaque colors translate into a strong belief that the sample belongs to that class, and more transparent colors indicate that such belief is smaller. With this matrix, the user can identify whether the model is confident toward the chosen class and at which point in the sequence process it achieves this confidence. It also allows the user to identify possible confusion among the labels in the dataset. Below the heatmap, we also summarize the class predictions to show the classes with the highest EP at a specific time step.

Visualizing the hidden state trajectory: The projection of the hidden states [Fig. 2(E)] also allows the user to inspect the evolution of an individual configuration, facilitating the analysis of how each time step impacts the hidden state and the EP. It also provides insight into when the model becomes sufficiently confident of its decision. Our tool adopts the concept of time series projections [26–29], which connects low-dimensional projections of points from a high-dimensional trajectory. Some of these approaches were designed to yield rather smooth curves [28, 29], for example, by including piecewise Bézier curves. However, our variant of the time series projections is purposely non-smooth (similar to the approach by Molchanov and Linsen [26] and van den Elzen et al. [27]) because we give the user a choice to specify an input and to visualize the specific points reached by the hidden state sequence produced by the input. We color code the trajectory according to the EP of the starting point of each line segment. Because the projection uses the same colors, it may sometimes be difficult to discern the trajectory. Hence, we provide the possibility of using gray levels instead to represent a temporal development. When showing a trajectory for a sequence, the transparency of all data points that do not belong to this sequence is increased. This technique addresses both the first and third challenges described in the previous section. This visualization and all other sequence-based views are linked to explore the relationship in different views when hovering over visual elements. Moreover, a tooltip for each element shows additional quantitative and text-based information about the classification up to the current word or the transition between two words.

Visualizing the distances between hidden states: In a bar chart, the Euclidean distances between hidden states are shown for the input sequences [Fig. 2(D)]. In particular, larger distances (which we also refer to as jumps) often push a classification further toward higher confidence in one specific class. They are usually associated with words that are common to the corresponding class. We provide a threshold such that each word associated with a jump is shown along the hidden state trajectory.

Visualizing words in the hidden state projection: It is optionally possible to show words that produce jumps between hidden states as an overlay on the hidden state projection. The corresponding words often correlate with words that are specific to one class. Similarly, the exploration of larger changes in time steps in the EP for specific classes might have a similar impact. Such changes are also visible in the input and heatmap visualization when the transparency of the color or the color itself changes. Using a threshold, words for these changes can also be shown on top of the projection. In addition, words for changes between classes (e.g., from positive to negative) can be added. Corresponding words may also be specific to a certain class because they change the expected classification up to this word. Often, the words may fall into multiple categories because they all express a change or make a large contribution to the prediction result. Showing these words for all sequences simultaneously for one of the categories also revealed some clusters. For example, words triggering larger changes for hidden states may have hidden states similar to other words, creating a similarly larger change.

Our visualization is sufficiently generic, it can be used in the analysis of different types of recurrent architectures, such as GRU [30] and LSTM [12].

Implementation: Data preparation and training were conducted using Python and TensorFlow [31]. Projections for t-SNE were generated using Scikit-learn [32]. Although all images in this paper were created using t-SNE, it would also be possible to use other projection methods instead. For our interactive visualization system, we generated a web interface implemented with JavaScript and Python; the visualizations were generated using D3.js [33]. The source code of our system is publicly available through DaRUS [17] and GitHub [18].

## Results and Discussion

To validate our visual analytics approach, we demonstrate some examples of how it can retrieve insight from models trained with two text classification datasets developed for sentiment analysis tasks, the IMDB dataset [34], and the Reuters dataset [25]. The IMDB dataset [34] comes with a binary classification problem in which every input is a text sequence containing a movie review that is either positive or negative. For this study, we used the IMDB dataset version available in Keras [13]. We trained a model containing a single LSTM layer [7] with 100 units and an output layer with a single sigmoid activation unit, achieving an accuracy of 85 %. We opted for a simple model because our goal is to visualize the impact of the recurrent layer in the model classification, and adding further layers would require including them in the analysis.

By contrast, the Reuters dataset [25] comprises thousands of articles on economics that can belong to one of more than 40 classes. For simplicity and easy visualization of the results, we trained our model using only the five most frequent classes (*grain*, *crude-oil*, *money-fx*, *acquisition*, and *earns*). For this task, we trained the model in a similar fashion as the previous model, using a single LSTM layer and an output layer containing five softmax output neurons, one for each class. After training, our model achieved a test-set accuracy of 93%.

Below, we demonstrate how our method can be used to conduct analytical tests in the aforementioned models. For the IMDB dataset, we used the first 100 sequences as input for our visualizations, and for the Reuters dataset, the first 500 sequences.

### Binary classification

Using our approach, we can explore the classification of binary data. In the following, we provide some insights into the IMDB dataset.

Classification overview: The overview visualization shows the relationship between the number of positive and negative reviews in the input data and how well they were classified [Fig. 1(B, C)].

Hidden state space: The 2-D hidden state projection allows users to analyze how the RNN models the high-dimensional hidden state space. In Fig. 1(D), we note that the model creates what seems to be a low-dimensional manifold embedded in the high-dimensional space, which continuously moves from completely negative reviews (pink) to completely positive reviews (blue).

Correct classification: Our tool facilitates an analysis of how the EP evolves through the sequence processing by displaying the trajectory of the configuration within the hidden state space and by displaying, with color encoding, the changes in the EP values. Figures 2 and 4 show the examples of correctly classified reviews. This includes typical reviews that were predicted as positive or negative with high certainty, reviews that jump between the two classes, and reviews in which it is not entirely certain throughout the prediction whether the classification should be entirely positive or negative.

Typical positive and negative classification results are shown in Fig. 4 for the images on the left. The trajectories in the hidden state projection start in a neutral region and then almost immediately jump toward the very positive or negative regions. This behavior is also visible in the heatmap visualizations.

Figure 4(c) shows an interesting insight uncovered by the tool for another sequence. Although the review is positive, it is written with several negative words appearing in most of the sequence, which makes the model alternate its hidden state between a positive and negative EP. Only by the end of the sequence does the model become certain that the review is indeed positive, and the hidden state converges to a region of a highly positive prediction.

Figure 4(d) shows an example of a review that is not very positive or negative. In between, although there are some rather positive or negative words, the review stays rather neutral overall, and the model struggles to classify toward a single direction. Because reviews can be also neutral with only a slight tendency toward positive or negative, such sequences may also be problematic for humans to clearly classify toward one direction.

By correlating the time steps with the hidden states created by them, our technique can identify undesired biases in the model. Biases occur when the decision process considers non-representative features that an expert does not consider if manually conducting the task. For instance, in Fig. 2(E), the EP of the model jumps to a highly positive value when the model reads the name of an actress, i.e., Kristy Swanson. This is not the desired behavior because there is nothing in that sentence up to that point that indicates a positive review, and ideally the model should only consider the sentiment of the review, and not whether a particular actor participated in the movie.

In all of these examples, some words are shown along the trajectories. These words are the result of the larger distances of hidden states when processing a sequence. Such distances for a whole sequence, for example, are visible in Fig. 2(D). A threshold (shown by the horizontal line) is used to show only words that result from a larger change. This visualization helps explore the strength of the change along the sequence. It also shows that the lengths of the lines in the projection do not always correlate with the distance in the original space owing to the nonlinear projection method applied. For example, this is visible when comparing the long blue line on the top in Fig. 2(E) to the pink line on the left. Although the pink line suggests a large jump, the histogram shows that the distance between the corresponding hidden states of the blue line is larger.

Incorrect classification: An example of an incorrect classification is shown in Fig. 5. Although this review was negative, it was misclassified as positive. An analysis of the visualizations showed that, at the beginning, the confidence toward a negative classification was higher. However, in the second half, the classification switches to a positive classification. This occurs because the name of an actor appears, and the last part refers to a different movie that was described with more positive words than the actual movie the review refers to. After the last words (academy award winner), the model has no chance to change the final prediction.

### Multi-class classification

In addition to a binary classification, our approach supports a multi-class classification. It can be applied similarly with some adaptation regarding the use of colors. Next, we report some insight for the Reuters dataset [25].

Classification overview: The classification overview (Fig. 6) shows the different classes with their corresponding color encoding, the number of test sequences available for each class, and how well the sequences were classified. It is clear that the frequencies for the classes vary substantially: *earns *and *acquisition* are frequently included, whereas *grain*, *crude-oil*, and *money-fx* are not. In addition, the correct classification achieved much better results for *earns*, *acquisition*, and *money-fx* than the others, which were mostly classified as *money-fx*. This provides some initial insight into the types of sequences that should be further analyzed.

**Fig. 7:**
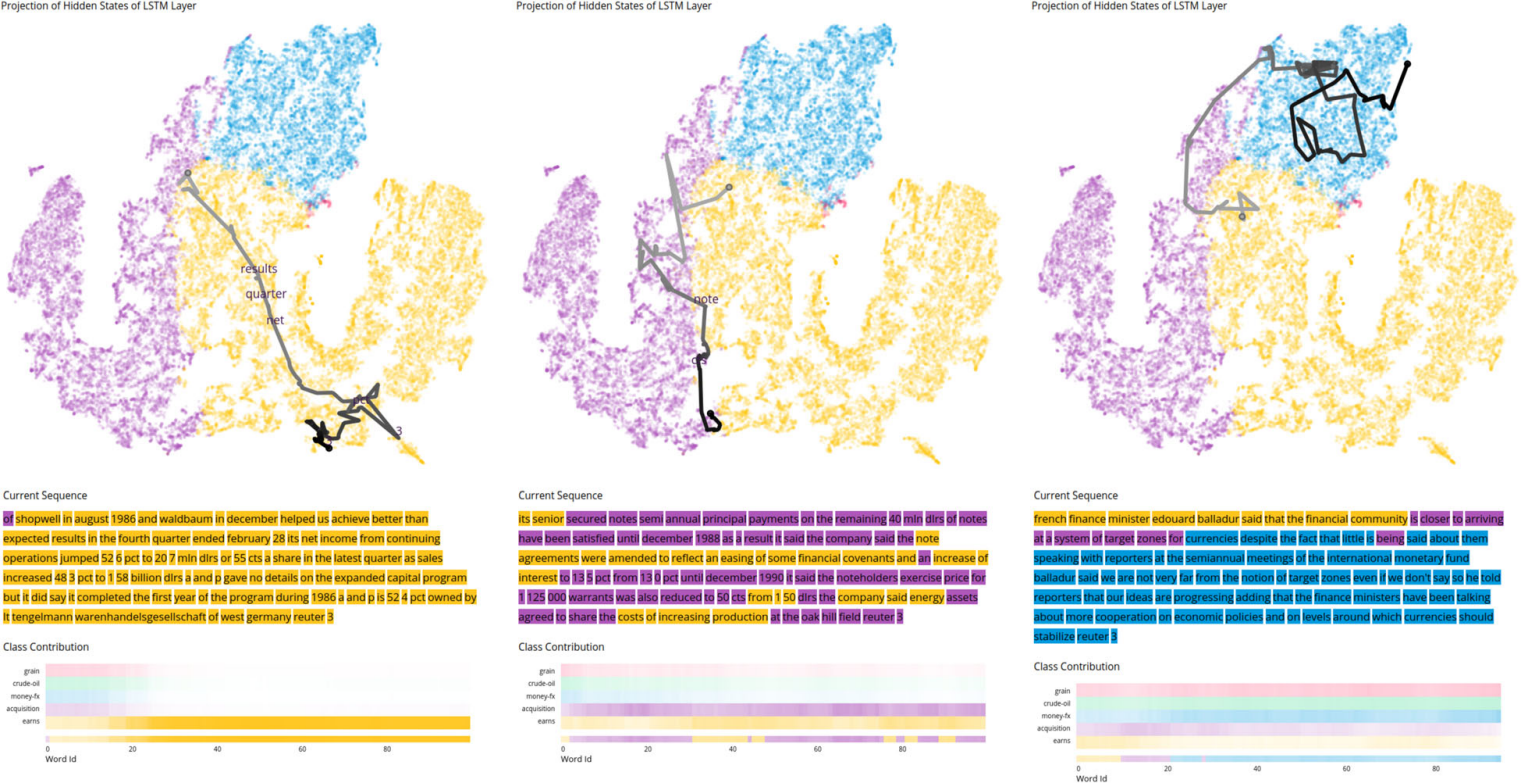
Examples of correctly classified sequences in a model trained for multi-class classification. The projection provides insight into the class distribution within the hidden state space. The heatmap matrix supports the analysis by displaying the evolution of the EP over sequence processing. Hence, the user can better identify at which time steps the model was able to distinguish between classes. In the visualizations on the left, the model is certain for the classification of *earns* (yellow). In the visualizations in the middle, the model is uncertain between *earns* (yellow) and *acquisition* (violet), and both classes have a similar EP while processing the sequence. In the visualizations on the right, the model is uncertain among all five classes and the EP for *money-fx* (blue) is only slightly larger compared to the other classes. Similar cases are likely to be misclassified. Underlying data source: Reuters [25]

Hidden state space and heatmap visualization: The hidden state projection visualization has certain limitations when handling multi-class classification models. In particular, it cannot properly display how each data point relates to each class. Here, the output of the model is a multidimensional vector in which each dimension represents the likelihood of the input being from a certain class. To mitigate this problem, we developed a supportive visualization in which data points are colored according to the more likely class generated by that hidden state. Hence, the color encoding in the projection simply represents the predicted class, without referring to the strength of the confidence in the prediction. Only the color encoding in the heatmap matrix shows the confidence in each individual class, using a color gradient ranging from white (low confidence) to the respective color (high confidence). In the projection visualization, it can be seen that the classes *earns*, *acquisition*, and *money-fx* dominate and build different regions (Fig. 7). To allow the user to visualize how the EP of a hidden state differs among the possible classes, the heatmap matrix visualization displays the evolution of the EP over the processing of an input sequence.

Correct classification: Fig. 7 shows multiple examples of correctly classified sequences from a model trained with the Reuters dataset. In Fig. 7 (left), we notice in the projection visualization that the hidden state sequence starts in a central location where classes are not clearly distinguishable. However, as the sequence processing progresses, the hidden state moves to a region with more certainty toward one of the classes [in this case, *earns* (yellow)]. This conclusion was supported by the heatmap matrix visualization. It should be noted that the model does not distinguish any class until time step 15. At this point, the model begins to converge toward the correct class. After the 20th time step, the model does not significantly change its EP until the end of the sequence, which leads us to believe that this sub-sequence contains sufficient information for the model to make a decision toward that class. Figure 7 (middle) shows an example in which the model is uncertain whether the sequence should be classified as *earns* (yellow) or *acquisition* (violet). This is visible in all views: The trajectory in the hidden state visualization moves along the border between the two corresponding areas of the different classes, the colors in the sequence switch between yellow and violet, and the heatmap visualization shows that both yellow and violet have higher confidence values compared to the other classes. Finally, Fig. 7 (right) shows an example in which the confidence for each class remains similar while processing the sequence. The correct class has only a slightly larger confidence value than the other classes. However, the trajectory clearly shows that all corresponding hidden states are located near other hidden states of the same classification.

**Fig. 8:**
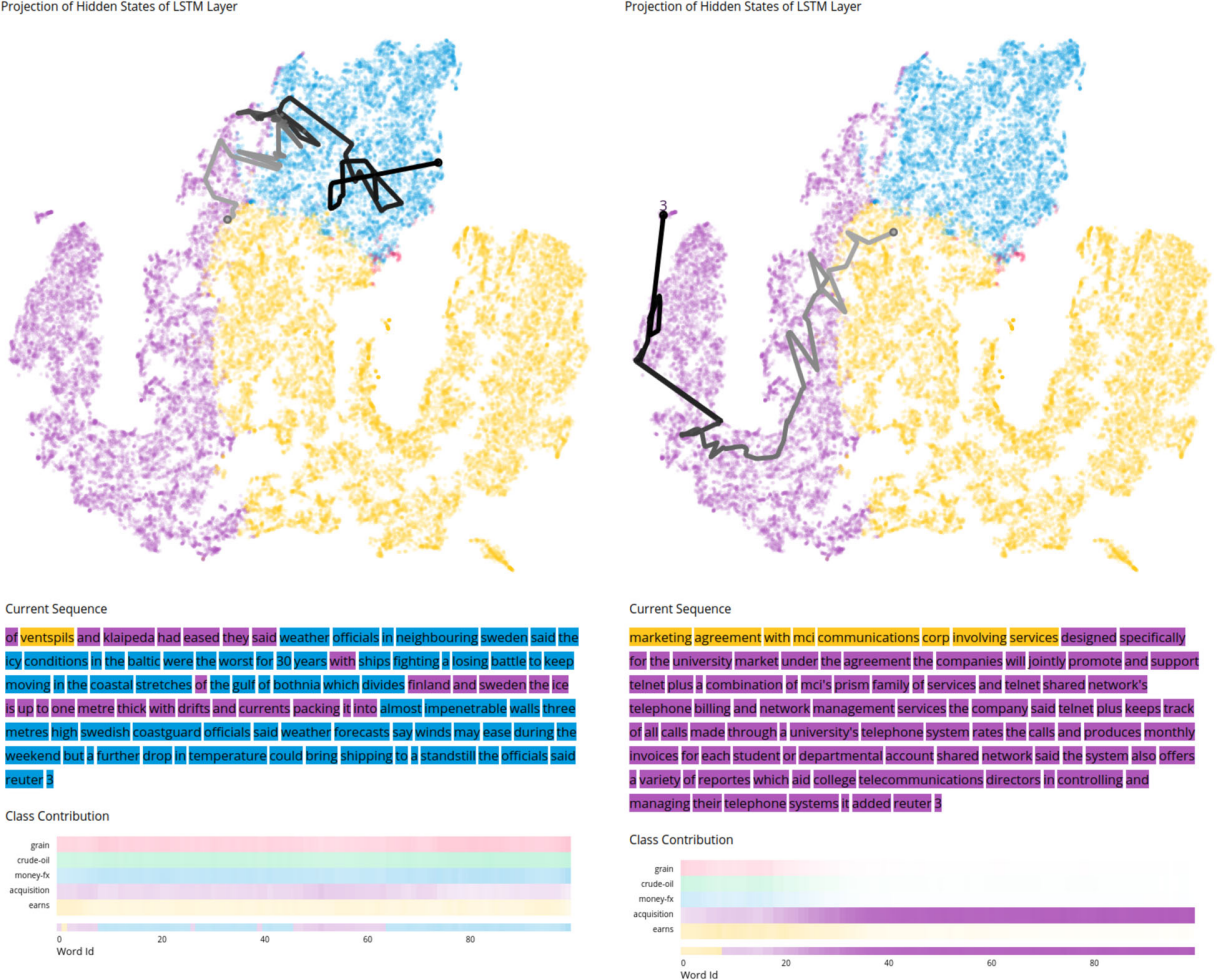
Examples of incorrectly classified sequences in a model trained for multi-class classification. The heatmap matrix supports the identification of the classes for which the model becomes confused and how the EP evolves throughout the training process, delivering an inaccurate result. In the left visualizations, the prediction is quite uncertain between all five classes. This is visible because the class contributions are similarly transparent for all classes. In the right visualizations, the model is rather certain in the classification of *acquisition* (violet), despite the correct class being *earns* (yellow). Underlying data source: Reuters [25]

Incorrect classification: Fig. 8 shows some examples of misclassifications. In Fig. 8 (left), an example similar to the previous one is visible, where the confidence for each class is similar. The heatmap matrix shows that the model never distinguishes the *earns* class (the correct one) from the others, and eventually chooses an incorrect class at the end of the sequence processing. This indicates that these classes are more difficult to identify using the model. In the projection, we can see that the trajectory is mostly located in the blue region (*money-fx* class). However, in Fig. 6, we can also see that there are many sequences incorrectly classified as *money-fx* (blue). This means that many samples in this region belong to a different class. This is also visible in Fig. 3, where we changed the colors in the projection to the actual classes of sequences instead of the classification results. At the top, where sequences were classified as *money-fx* (blue), the classes should actually be *crude-oil* (green) or *grain* (red) in many cases.

Figure 8 (right) shows an example in which the model is more certain toward a specific class [*acquisition* (violet)]. However, the correct class is *earns* (yellow), which has a higher confidence only at the beginning of the sequence. When manually comparing different input sequences, it is sometimes also extremely difficult for humans to differentiate classes and correctly classify them. Multiple classes are also occasionally appropriate. The fact that *earns* more often contains digits compared to *acquisition* that more often contains continuous text, might be one possible explanation for the improper classification of this example.

## Conclusions

In this paper, we introduced a visual analytics approach to address three interpretability challenges in the analysis of RNNs trained for NLP applications. We demonstrated through use cases some practical insights that one can achieve from using our techniques and that can be instrumental in improving the interpretability of these models.

As a topic that has attracted strong interest in both the ML and visualization research fields, there are certainly more findings to come in the future. Notably, it would be interesting to see more research on interpretability issues from models addressing particular types of input data, such as videos [35], financial data [36], and eye tracking [37]. Interpretability is the main bottleneck faced by deep learning models, and more innovation in this topic will undoubtedly bring more improvements across many different applications.

## Data Availability

Both the IMDB and Reuters dataset are available in Keras [13]. The source code of our system is publicly available through DaRUS [17] and on GitHub [18].

## Abbreviations

RNN: Recurrent neural network
t-SNE: t-distributed stochastic neighbor embedding
LSTM: Long short-term memory
NLP: Natural language processing
ML: Machine learning
DNN: Deep neural network
EP: Expected prediction
